# Impact of a bathing tradition on shared gut microbe among Japanese families

**DOI:** 10.1038/s41598-019-40938-3

**Published:** 2019-03-13

**Authors:** Toshitaka Odamaki, Francesca Bottacini, Eri Mitsuyama, Keisuke Yoshida, Kumiko Kato, Jin-zhong Xiao, Douwe van Sinderen

**Affiliations:** 1 Next Generation Science Institute, Morinaga Milk Industry Co., Ltd, Zama, Kanagawa Japan; 2 APC Microbiome Ireland and School of Microbiology, National University of Ireland, Western Road, Cork, Ireland

## Abstract

Sharing of *Bifidobacterium longum* strains had recently been shown to occur among Japanese family members, a phenomenon that is not confined to mother-infant pairs. In the current study, we investigated if bathtub water is a possible vehicle for the exchange of strains as a consequence of a Japanese custom to share bathtub water by family members during bathing practices. A total of twenty-one subjects from five Japanese families, each consisting of parents with either 2 or 3 children, were enrolled in this study and the fecal microbiota of all participants was determined. Viable bifidobacterial strains were isolated from all bathtub water samples. A subsequent comparative genome analysis using ninety-eight strains indicated that certain strain-sets, which were isolated from feces and bathtub water, share near identical genome sequences, including CRISPR/Cas protospacers. By means of unweighted UniFrac distance analysis based on 16S rRNA gene analysis of 59 subjects from sixteen Japanese families, we showed that the fecal microbiota composition among family members that share bathtub water is significantly closer than that between family members that do not engage in this practice. Our results indicate that bathtub water represents a vehicle for the transmission of gut bacteria, and that the Japanese custom of sharing bathtub water contributes to the exchange of gut microbes, in particular bifidobacteria, among family members.

## Introduction

Initial bacterial colonization and the establishment of an intestinal gut microbiota starts at birth, where passage through the birth canal facilitates the first bacterial transfer event between mother and newborn^1^. A number of studies have provided convincing evidence to support vertical transmission of gut microbes from mother to newborn at the very early stages of life. For example, Makino *et al*. revealed that vaginally delivered infants share at least one monophyletic strain belonging to the genus *Bifidobacterium* with their mothers, whereas this strain-sharing phenomenon was not observed among infants delivered by C-section^2^. Asnicar *et al*. have shown that several specific strains, including those belonging to *Bifidobacterium bifidum*, *Coprococcus comes*, and *Ruminococcus bromii*, are present in samples from corresponding mother-infant pairs, while being clearly distinct from those carried by non-corresponding pairs^3^. A recent comparative genome analysis showed no difference between vaginal- and gut-derived bifidobacterial strains, thus enforcing the importance of the maternal vaginal microbiome as a source of (certain elements of the) infant gut microbiota^4^. Furthermore, a recent study based on strain-level metagenomic profiling provided evidence for vertical microbial transmission from multiple maternal sources, with the vaginal, skin, oral, and gut microbiome to the early infant communities^5^. This work also showed better persistence of maternal gut strains in the infant gut as compared to those transmitted from other sources.

Nonetheless, vertical transmission of gut microbes from mother to infant only partially explain the composition of our gut microbiota which also changes with aging^6^. Strain-level analyses have recently confirmed that a significant fraction of the developing microbiome is indeed of maternal origin, though this seeding event is selective, and strains from certain phyla are also acquired from the environment^7^. In a previous study, we identified bifidobacterial strains which appear to have been shared across three generations of family members, based on the very high similarity observed between the genome sequences of bifidobacterial isolates^8^. Furthermore, it is noteworthy that we observed that such apparent sharing events had not only occurred between mother and corresponding child pairs, but also between father and child, or between husband and wife. Indeed, some studies have suggested the notion of postnatal gut colonization mediated by the environment, which continues into adulthood^9,10^, though the precise transmission route is still a matter of speculation.

Since intra-family sharing of bacteria is an observed phenomenon^9,10^, one of the plausible transfer vehicles is constituted by the family home. Lax *et al*. have reported that each home has its own microbiome, which is largely being sourced from humans^11^. Furthermore, a bacterial ecology study of house dust found that certain bacterial taxa of this niche are also members of the gut microbiota^12^. These findings imply that bacterial transfer may indeed commonly occur within the family home, although precise details of such presumptive strain transmission are currently not known.

Bathing at home is a practice that intends to improve personal hygiene and eliminate body odors, but can also be associated with religious rituals or serve therapeutic purposes. The purpose of bathing within the Japanese culture is to promote relaxation and/or communication and bonding between family members (e.g. parent and child), in addition to improving personal hygiene. According to this Japanese custom, someone has to clean oneself with soap and rinse before entering the bathtub, so as not to contaminate the bath water. Therefore, Japanese commonly don’t change the bathtub water until the last family member has finished bathing.

The aim of the present study was to investigate if bathtub water represents an environmental vehicle that facilitates exchange of bifidobacterial strains between family members, as a consequence of a Japanese custom to share bathtub water during bathing practices.

## Results

### Comparison of bathtub and fecal microbiota

**Figure 1:**
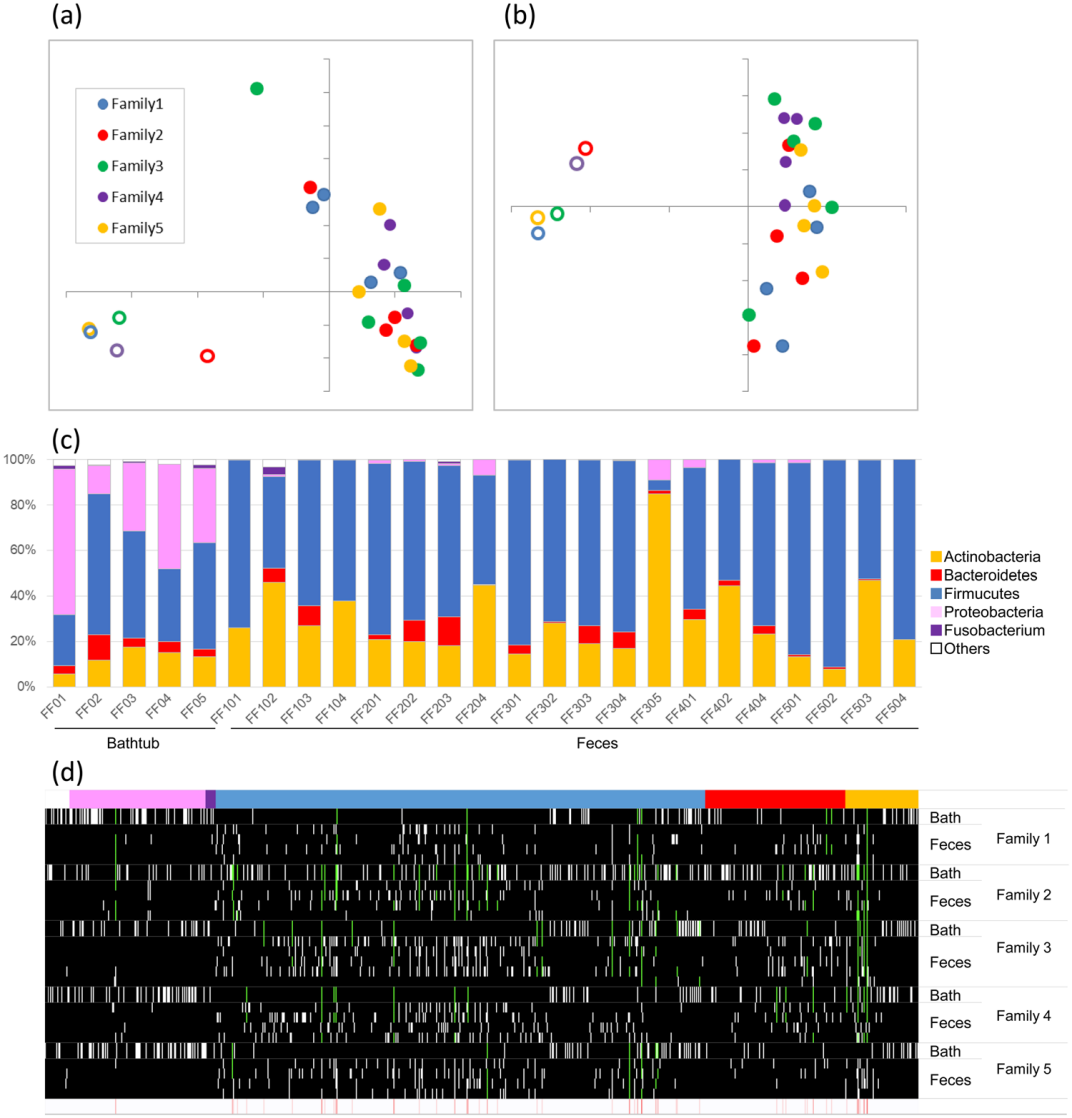
Microbiota in bathtub water and fecal samples. (**a**) Weighted and (**b**) Unweighted UniFrac PCoA of bathtub and fecal microbiota from five family members. Open and closed symbols indicate bathtub microbiota and fecal microbiota, respectively. (**c**) Composition of bathtub and fecal microbiota at phylum level. Each sample ID is shown in Supplementary Table 1. (**d**) Matrix of OTUs shared with the bathtub and fecal samples in each family. Horizontal and vertical columns indicate OTU and sample, respectively. Colours in the top horizontal column represent the OTU-associated phylum as shown in (**c**). White and black colours in the matrix represent presence and absence of each OTU, respectively. Light green indicates OTUs shared with the bathtub and fecal samples in each family. Bottom red-white heatmap shows number of families possessing the OTUs shown as light green.

We first investigated by PCR if bacteria were present in two samples of bathtub water prior to any family member taking a bath (family ID01 and ID05, see Supplementary Table 1). The results were negative, indicating that the bacterial cell number was below the detection limit (<10 cells per one liter). Therefore, we presumed that bacteria detected in bathtub water (following family members taking a bath) had originated from any of these family members. A total of 207,129 high-quality paired sequences were obtained from 5 samples of used bathtub water and 21 Japanese subjects, with 7,967 ± 1,607 high quality reads per sample (Supplementary Table 1). As expected, fecal microbiota and that of bathtub water were shown to be distinctly different (Fig. 1a,b). The composition of fecal microbiota included four predominant phyla in agreement with previous results^6^, whereas bathtub water microbiota was composed of a higher proportion of Proteobacteria (Fig. 1c). Based on our observation, individual bathtub water microbiota assessments were clearly distinct, where only 24.55 ± 5.46% of identified OTUs were matched to OTUs derived from other bathtub water samples (Fig. 1d). Notably, 5.66 to 18.24% of OTUs retrieved from bathtub water samples per family were shown to correspond to OTUs derived from fecal microbiota samples of each family member, although these common taxa constitute a minority of the total bathtub water microbiota, whereas major components are predicted from the environment^11^ and skin^5,13^, such as Proteobacteria, *Staphylococcus* and *Streptococcus* (Fig. 1d, Supplementary Table 2). In total, 52 of the 442 identified OTUs in bathtub samples, and all belonging to the 4 major phyla of gut microbiota, Firmicutes, Bacteroidetes, Actinobacteria and Proteobacteria, were shared with those present in fecal samples. Of these, 44 OTUs were assigned to obligate anaerobes. Notably, despite the fact that bifidobacteria constitute an underrepresented bacterial group in bathtub water, the same OTUs taxonomically assigned to this genus were widely distributed in both fecal and corresponding bathtub water samples.

### Isolation of strains

In order to investigate if (some of) the identified bathtub microbes are viable, we isolated bifidobacterial strains as a representative microbial group that is both found in the feces and as well as in bathtub water. We succeeded in isolating dozens of viable bifidobacterial strains from all tested bath water samples. The number of colony forming units (CFUs) per 3 liters bath water varied from 12 to 1,701 on TOS agar medium. A PCR-based approach with a genus-specific primer pair showed that 540 out of 669 assessed colonies were identified as bifidobacteria. We then randomly selected between 1 and 7 individual isolates from each sample (from both bathtub water and feces) for genome sequence analysis following an initial RAPD analysis in order to remove suspected clonal strains isolated from the same sample (Supplementary Table 1). In this manner, genome sequences were generated for 98 strains. Taxonomical classification of all isolates was confirmed by 16S ribosomal RNA sequence and phylogenetic analysis performed on the *Bifidobacterium* core genome (Supplementary Fig. 1 and Supplementary Table 3).

### General features of *Bifidobacterium* genomes

General features of each of the newly determined *Bifidobacterium* genomes are presented in Supplementary Table 3. Sequencing generated between 8 and 127 contigs for each strain. The predicted genome size ranged between 2.11 Mb and 2.67 Mb, possessing an average predicted ORF number of 1,975 per genome, of which the longest species average was 2.44 ± 0.11 Mb for *B. longum* subsp. *longum*, wheares the shortest species average was found to be 2.25 ± 0.11 Mb in the case of *B. adolescentis*. We also identified CRISPR-Cas systems on the genome of sixty-one of our isolated strains. These CRISPR-Cas systems were shown to be representatives of the II-C, I-C, I-U, I-E and II-A subtypes, possessing between 5 and 76 protospacers (Supplementary Tables 3 and 4).

### Comparative genomics

**Figure 2:**
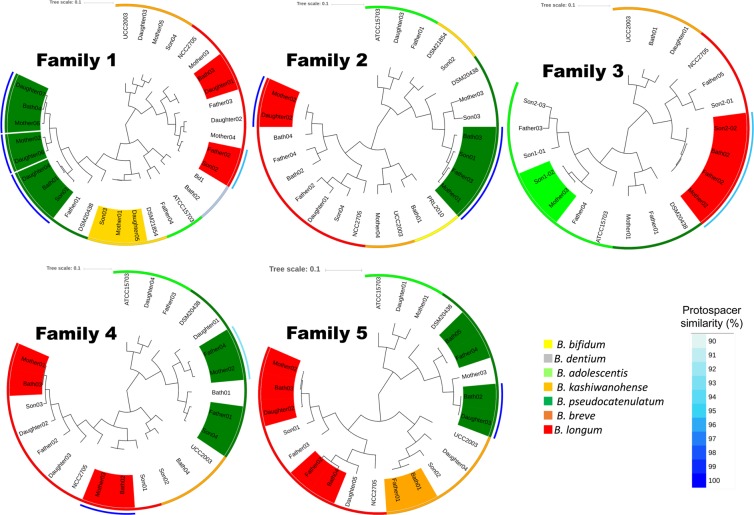
MCL clustering of bifidobacterial strains isolated from bathtub water and feces. MCL hierarchical clustering based on the presence/absence of open reading frames (ORFs) of 98 novel bifidobacterial isolates obtained from five families, compared with the ORF content of 7 publicly available *Bifidobacterium* representatives. Strain-pairs with a similar color code indicate a nearly identical gene content and genome sequence, with ANI values of more than 99.5% (as in the case of monophyletic strains). The circles, from outside to inside, indicate CRISPR similarity, where present, and species.

MCL clustering was conducted for each family to predict the origin of isolates from bathtub water (Fig. 2). Among 19 strains isolated from bathtub water, 12 strains were shown to contain a near identical gene content with a fecal isolate from a corresponding family member. Each highlighted strain-pair in Fig. 2 was shown to display a very high level of sequence identity (Supplementary Fig. 2) including the protospacers of CRISPR-Cas systems, where present (Supplementary Table 4). Seven pairs of these strains possess a CRISPR-Cas system with fully matching protospacers, whereas in three strain pairs, the region of protospacers in the CRISPR-Cas system was partially different. Notably, all 19 strain pairs from 5 family members were shown to be more than 99.5% identical (Average Nucleotide Identity or ANI value), which value is consistent when dealing with monophyletic strains. These findings imply that such bath water-derived strains had originated from gut microbes. We also found twelve strain-pairs which had been retrieved across family members. These pairs were not confined to *B. longum* subsp. *longum*, as had been reported previously^8^, but also involved *B. pseudocatenulatum*, *B. breve* and *B. kashiwanohense* (Fig. 2).

### Bathtub isolates in fecal samples

Furthermore, we evaluated the cell number of certain isolates from bathtub water in fecal samples and possibly transmitted strains among family members by strain-specific PCR. A total of 13 strain-specific primer pairs were thus designed for strains possessing a CRISPR-Cas system, based on the protospacer-sequences (Supplementary Table 5). Of 11 strains detected in one or more fecal samples, cell numbers were estimated to range between 10^5^ and 10^9^ bacteria per gram (Supplementary Table 6). It is worth mentioning that our isolation efforts resulted in the identification of 98 strains, whose distribution across samples as assessed by strain-specific PCR appears to be wider than what was observed solely based on cultivation. For example, no F02Bath01 derivatives were isolated from feces of any family2 member, however, qPCR results indicate the presence of this strain (or a very close relative) in three members of this family. Similarly, the presence of apparently clonal F04Bath02 strains in all family4 members was highlighted by qPCR, though we could only isolate this strain from one family member (FF402). In addition to this we also assessed the survivability of strains in 40-degree hot water. Interestingly, no difference was observed in survivability of bathtub isolates compared with type strains of bifidobacteria, when tested in hot water after 60 and 120 minutes (Supplementary Table 7).

### Similarity of fecal microbiota between family members taking a bath together

**Figure 3:**
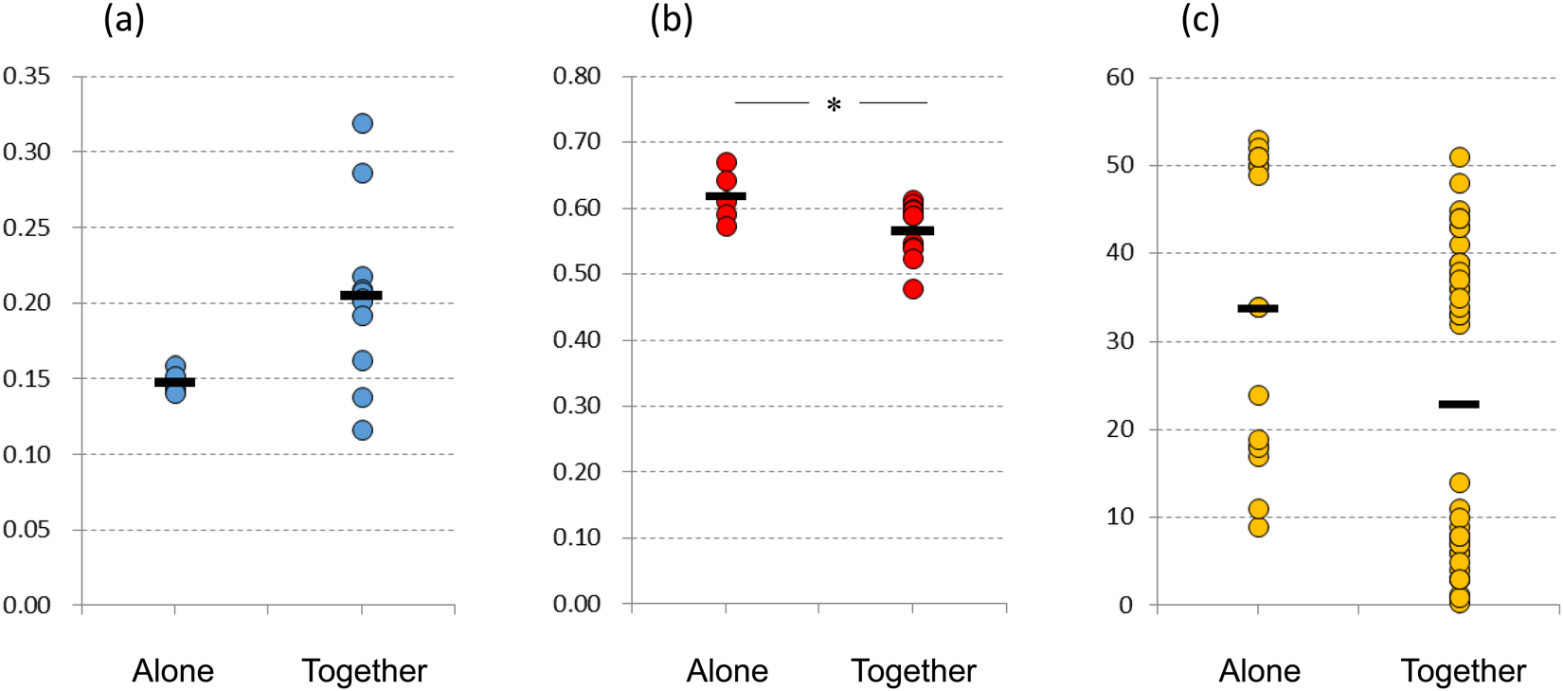
Comparison of fecal microbiota similarity between family members taking a bath together (together group) and family members taking a bath alone (alone group). Weighted UniFrac distance of fecal microbiota between family members based on 16S rRNA gene analysis. This distance, representing the abundance of observed OTUs, reflects the similarity of the gut microbiota composition between subjects. (**b**) Unweighted UniFrac distance of fecal microbiota between family members based on 16 S rRNA gene analysis. This distance, based on presence or absence of each OTU, reflects the number of OTUs shared between subjects. Weighted and Unweighted UniFrac distances were analyzed by the unpaired t-test. (**c**) Subject age in each group.

In order to investigate the impact of a ‘bathing together’ custom on the mutual exchange of gut microbes between family members, we also compared the similarity of fecal microbiota between 11 families. The family members were divided into two groups: members taking a bath together at the same time (together group) and members sharing the same bathtub water, but taking a bath alone for more than a year (alone group). Weighted UniFrac distance of fecal microbiota between members of the together group tended to be higher (p = 0.051) than that between alone group (Fig. 3a, Supplementary Table 8), possibly due to the larger difference of subject age between family members (Fig. 3c). Nevertheless, the result of unweighted UniFrac distance was significantly *vice versa* (p = 0.037, Fig. 3b, Supplementary Table 9), indicating that the number of gut microbes shared by family members of the together group was higher than that in the alone group. This result thus corroborates the notion that bathtub water is a genuine environmental vehicle that supports microbial exchange of bifidobacterial strains and that the Japanese custom of sharing bathtub water contributes to the exchange of gut microbes across family members.

## Discussion

Gut microbiota studies have seen a rapid expansion in recent years, however, the mechanisms underlying the establishment and interaction between members of the gut community are still obscure and under active investigation. The precise transmission routes of gut microbes are still debated, and our work has revealed a heretofore undiscovered environmental factor contributing to the exchange of bifidobacterial strains, involving the Japanese custom of sharing bathtub water by family members.

Microbiota analysis revealed that about 10% of bathtub microbes per family matched OTUs obtained from the fecal microbiota of each family member (Fig. 1d). Notably, our analysis revealed that certain bifidobacterial strain pairs, which were isolated from feces and bathtub water of members of the same family were also monophyletic (Fig. 2). In addition, no monophyletic bifidobacterial strains were isolated from subjects belonging to different families. Of course our analysis does not exclude the possibility that a detected strain was already present in the gut microbiota of subjects irrespective of the bath-taking practice. To counter this possibility, we calculated unweighted UniFrac distance, which showed that the similarity of fecal microbiota between family members taking a bath together (together group in Fig. 3) was significantly higher than that between family members taking a bath alone (alone group in Fig. 3), indeed supporting the idea that bathtub water is one of the possible environmental vehicles that explain how gut microbes are being shared among (a subsection, depending on how common this bathing practice is, of) the Japanese population.

It is noteworthy that bifidobacteria were frequently found in both fecal and bathtub water samples (Fig. 1c,d). The bifidobacterial strains isolated from bathtub water are expected to represent, at least partially, those present in the gut microbiota of family members as based on comparative genomics and the real-time PCR results, though not all of them were confirmed to be shared between family members (Fig. 2, Supplementary Table 6). Notably the fact that some of the strains could not be detected across members of the same family does not necessarely exclude that in some cases they could have been first acquired and subsequently lost or they simply could not be retrieved by our isolation efforts.

It has been reported that the Japanese gut harbors a higher proportion of bifidobacteria compared to individuals from other countries^6,14,15^. This may be due to a variety of factors, such as uniqueness of the Japanese diet^16^. Our results suggest that a common Japanese bathing custom may also contribute to this relatively high bifidobacterial abundance. Furthermore, our study suggests that strain sharing via bathtub water may not be restricted to bifidobacteria, as subjects with the custom of taking a bath together seem to possess a more similar gut composition (Fig. 3b). In line with this notion is our finding that 52 OTUs in bathtub water, which belong to either of the 4 major phyla of gut microbiota, were also present in the corresponding fecal samples (Supplementary Table 2).

The result of unweighted UniFrac distance also showed the impact of the Japanese custom of sharing bathtub water between family members on their gut microbiota (Fig. 3b). Also, if we consider that the bathtub water represents a microaerophilic environment, it is surprising that some of anaerobic gut microbes (such as bifidobacteria) still remain viable (Supplementary Table 7). As some of the anaerobic gut microbes lost viability after 60 minutes exposure to 40 °C water (Supplementary Table 7), one would think that Japanese subjects taking a bath alone (alone group in Fig. 3) should have less chance of acquiring gut microbes from bath water. In this case the similarity of gut microbiota between Japanese subjects taking a bath alone may be closer to that of subjects from a non-Japanese population (assuming that they do not share bath water) as compared with that of Japanese subjects taking a bath together. Unfortunately, comparing the similarity of gut microbiota between Japanese individuals with those from other countries using the results obtained from this study and other studies is unlikely to be informative due to differences in the employed methodologies, especially DNA extraction and utilized primers^17^. Large-scale and global population studies combined with the establishment of a standard protocol for human fecal sample processing in metagenomic studies^17^ will be needed to properly address this question.

## Conclusions

We revealed a plausible vehicle for the exchange of gut microbes for Japanese subjects. Notably, our findings reveal that transmission of gut microbes is not only restricted to mother-child pairs, but can have a bi-directional way and the Japanese bathing custom may promote such strain transmission. The result of this study provides a first glimpse as to how environmental factors and cultural habits (other than diet) may influence gut colonization and this should be taken into consideration for future studies investigating factors that affect gut microbiota composition.

## Methods

### Sampling of bathtub water

Five Japanese families, each consisting of two parents with either 2 or 3 children, were enrolled in this study. Four liters of bathtub water were collected once using sterilized pet bottles within 30 min before (2 families) and after family members (5 families) took a bath, then microbes were concentrated by suction filtration using four pieces of Isopore Membrane filters HTTP04700 (Millipore, Bedford, Massachusetts, USA) with a diameter of 47 mm and a mesh size of 45 µm. One part of the filter, which subsequently was subjected to 16S rRNA gene-based microbiota analysis, was stored at −20 °C until analysis. The remainder of the filter was put onto a TOS propionate agar plate (Eiken Chemical, Tokyo, Japan) supplemented with 50 mg/l mupirocin (Merck KGaA, Darmstadt, Germany), which is a selective medium for bifidobacteria^18^, and was then immediately enclosed in plastic bags containing AnaeroPouch (Mitsubishi Gas Chemical, Tokyo, Japan) to create an anaerobic environment so as to retrieve bifidobacterial strains. These plates were anaerobically kept at room temperature and were then transported to the laboratory within 16 hours for cultivation at 37 °C.

### Microbiota analysis

A total of 59 fecal samples (Supplementary Tables 1 and 10), collected once from each subject and subsequently stored at −80 °C, were obtained as part of a previously described cross-sectional study^6^. The study was approved by the ethics committee of Kensyou-kai Incorporated Medical Institution (Osaka, Japan). Written informed consent was obtained from all subjects or their legal guardians. All methods were performed in accordance with the relevant guidelines and regulations.

A piece of filter used for collecting microbes in bathtub water was cut randomly, and was then suspended in 450 μl of extraction buffer (100 mM Tris-HCl, 40 mM ethylene diamine tetra acetic acid, pH 9.0) and 50 μl of 10% sodium dodecyl sulfate. 20 mg of a fecal sample was also suspended in the same extraction buffer. Subsequent DNA extraction and sequencing by Illumina Miseq (Illumina, Inc., San Diego, CA, USA) were performed as described previously^8^. After removing sequences consistent with data from the Genome Reference Consortium human build 38 (GRCh38) and phiX reads from the raw Illumina paired-end reads, the sequences were analyzed using the QIIME2 software package version 2017.10 (https://qiime2.org/). Potential chimeric sequences were removed using DADA2^19^, followed by trimming 30 and 90 bases of the 3′ region of the forward and the reverse reads, respectively. Taxonomical classification was performed using Naive Bayes classifier trained on the Greengenes13.8 with a 99% threshold of OTU full-length sequences. UniFrac distances were calculated using QIIME2 software (Supplementary Tables 8 and 9) and were shown as average score at each family in Fig. 3.

### Isolation and identification of strains

Three pieces of filter, with a diameter of 47 mm, were first placed on a TOS propionate agar plate, and were then removed, followed by incubation at 37 °C for 48 hours under anaerobic conditions. Twelve to sixty-three colonies per sample (see Supplementary Table 1) were randomly selected and were examined by colony PCR using a primer set specific for the genus *Bifidobacterium*, as based on 16S ribosomal RNA gene sequence (g-Bifid-F: 5′-CTCCTGGAAACGGGTGG-3′; g-Bifid-R: 5′-GGTGTTCTTCCCGATATCTACA-3′)^20^. Each identified bifidobacterial isolate was cultivated in Difco Lactobacilli MRS (Becton Dickinson, NJ) supplemented with 0.05% L-cysteine HCl (Kanto Chemical, Tokyo, Japan) at 37 °C for 16 hours under anaerobic conditions before DNA extraction. A total of 669 strains were isolated and assessed by random amplified polymorphic DNA (RAPD) analysis to detect (and, in case they appeared to be same, discard) clonal isolates obtained from the same sample. In this manner, 98 distinct isolates were selected for genome sequencing.

### Genome analysis

Genomic DNA extraction, library construction for an Illumina MiSeq instrument and subsequent *de novo* assembly of raw reads by the CLC Genomics Workbench (v 8.0) software package (Qiagen, Valencia, CA) were performed as previously described^8^.

Open Reading Frame (ORF) prediction was performed using PRODIGAL (version 2.6; http://prodigal.ornl.gov/) and supported by BLASTX v2.2.26^21^ alignments. Automatic annotation was performed as described previously^8^ and based on BLASTP v2.2.26 alignments^21^ and the non-redundant protein database curated by the National Centre for Biotechnology (http://www.ncbi.nlm.nih.gov/). Where necessary, manual editing was performed using Artemis v.15^22^ which was employed for output visualization. Where relevant, annotations were further refined using a combination of protein family (Pfam)^23^ and COG^24^ databases. Transfer RNA genes were identified using tRNAscan-SE v1.4^25^.

### Comparative genomics and Phylogenetic analysis

Seven strains (Supplementary Table 11), which belong to the same species as the isolates described in the current study, were used for comparative purposes. Single core gene families (GFs), being present in all of the seven strains and 98 isolates, were identified (Supplementary Table 12). Comparisons of amino acid sequences were performed using an all-against-all, bi-directional BLAST alignment (cut-off: E-value 0.0001, with at least 50% identity across at least 50% of either protein sequence), followed by clustering into protein families using the Markov Cluster Algorithm (MCL) implemented in the mclblastline pipeline v12-0678^26^. Distances were based on the covariance value and were calculated for the hierarchical clustering analysis using MeV suite Version 4.9 (https://sourceforge.net/projects/mev-tm4/). Genomic alignments were conducted using the progressive Mauve tool with default settings^27^. Phylogenomic inference was performed based on the alignment of a set of single core GFs as described previously (Supplementary Fig. 1)^8^.

### Strain-specific PCR

A specific primer-pair was designed for each strain that had been isolated from bathtub water, as based on identified protospacers (Supplementary Table 5) and following detection of clustered regularly interspaced short palindromic repeats (CRISPR)-*cas* systems by CRISPR finder (http://crispr.i2bc.paris-saclay.fr/Server/). The specificity of the designed primers was confirmed both by BLASTN alignments performed against all genomes in this study, and by PCR assays using DNA samples extracted from all isolates and fecal samples as positive or negative controls (Supplementary Table 13). Real-time PCR was conducted using a Smart Cycler II system (Cepheid, Sunnyvale, California, USA) and SYBR Premix Ex Taq (TaKaRa Shuzo) to determine fecal cell numbers of strains corresponding to those isolated from bathtub water. The amplification program consisted of 1 cycle of 94 °C for 10 seconds, followed by 40 cycles of 94 °C for 5 seconds and 60 °C for 30 seconds. DNA extracted from each bathtub isolate, for which corresponding cell numbers in culture broth were determined by microscopy, was used to produce a standard curve.

### Statistical analysis

Weighted and Unweighted UniFrac distance of fecal microbiota between members were analyzed by the unpaired t-test using SPSS version 22.0 statistical software (IBM Corp., Armonk, NY, USA). For all statements, p-value of < 0.05 was considered statistically significant.

### Ethics approval and consent to participate

The study was approved by the ethics committee of Kensyou-kai Incorporated Medical Institution (Osaka, Japan). Written informed consent was obtained from all subjects or their legal guardians.

## Supplementary information

Supplementary Information

Supplementary Table 1-13

## Data Availability

DNA sequences corresponding to 16S rRNA gene data were deposited in DDBJ under accession numbers DRA007021 (Supplementary Tables 1 and 10). Genome sequences of bifidobacteria were submitted to GenBank (accession numbers are listed in Supplementary Table 3). The datasets used and/or analyzed during the current study are available from the corresponding author on reasonable request.
