# Author Correction: Impact of a bathing tradition on shared gut microbes among Japanese families

**DOI:** 10.1038/s41598-020-59023-1

**Published:** 2020-02-05

**Authors:** Toshitaka Odamaki, Francesca Bottacini, Eri Mitsuyama, Keisuke Yoshida, Kumiko Kato, Jin-zhong Xiao, Douwe van Sinderen

**Affiliations:** 1 Next Generation Science Institute, Morinaga Milk Industry Co., Ltd, Zama, Kanagawa Japan; 2 APC Microbiome Ireland and School of Microbiology, National University of Ireland, Western Road, Cork, Ireland

Correction to: *Scientific Reports* 10.1038/s41598-019-40938-3, published online 13 March 2019

The original version of this Article contained an error in the title of the paper, where the word “microbes” was incorrectly given as “microbe”. This has now been corrected in the PDF and HTML versions of the Article and in the accompanying Supplementary Information files.

