# Supplementary material for: Impact of a bathing tradition on shared gut microbe among Japanese families
Source: Sci Rep. 2019 Mar 13;9:4380. doi: 10.1038/s41598-019-40938-3 (PMC6416414; doi:10.1038/s41598-019-40938-3)
Supplement: Supplementary file 1 — Supplementary Information [file 41598_2019_40938_MOESM1_ESM.pdf]

-Supplementary Information-

Impact of a bathing tradition on shared gut microbe among Japanese families

Toshitaka Odamaki[1,2*], Francesca Bottacini[2], Eri Mitsuyama[1], Keisuke Yoshida[1], Kumiko Kato[1], Jin-zhong Xiao[1] and Douwe van Sinderen[2]

[1]Next Generation Science Institute, Morinaga Milk Industry Co., Ltd., Zama, Kanagawa, Japan

[2]APC Microbiome Ireland and School of Microbiology, National University of Ireland, Western Road, Cork, Ireland

Inventory of Supplementary Information

1. Supplementary Figures and Legends
   Supplementary Figure S1-S2

2. Supplementary Tables
   Supplementary Table S1-S13

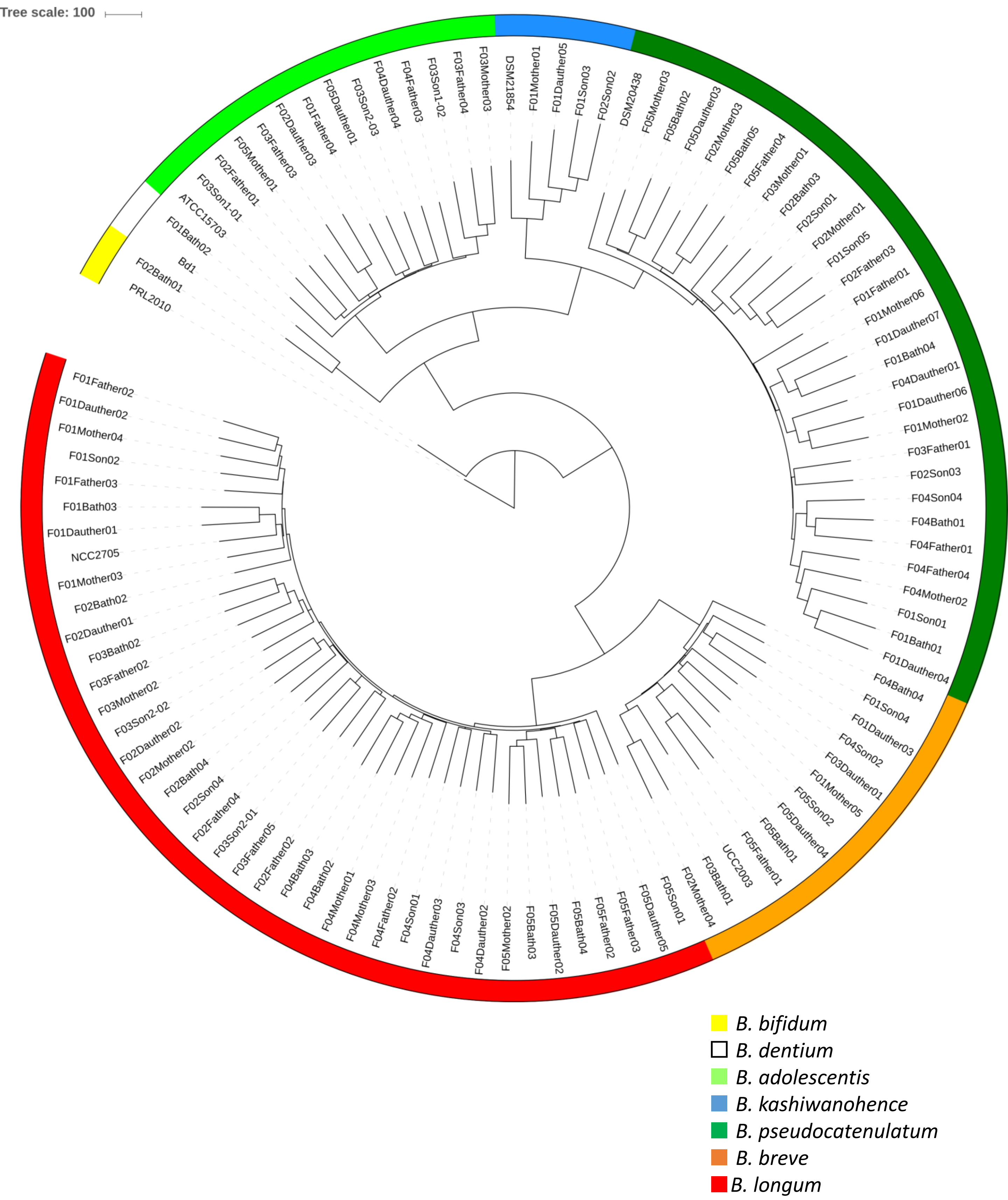

Supplementary Fig. 1

Cladgram of Bifidobacterial strains

We identified taxonomical classification of all isolates by the phylogenetic analysis performed on the *Bifidobacterium* core genome.

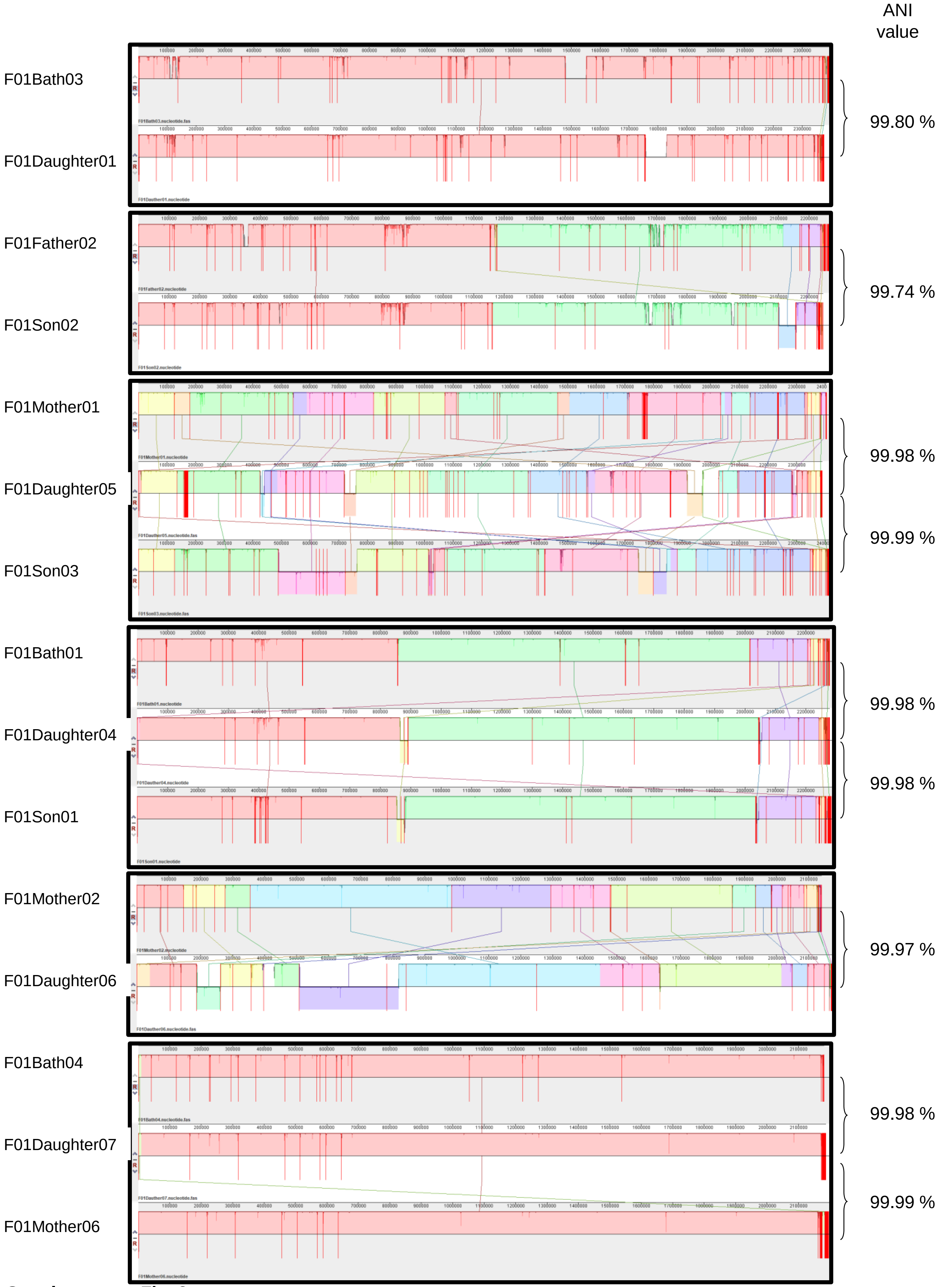

Supplementary Fig. 2a

Sequence similarity between strains putatively transmitted among family1 members and bathtub water

Related to Figure2. As shown in Mauve whole nucleotide alignment, certain strain-sets which were isolated from feces and bathtub water share near identical genome sequences, suggesting that bifidobacterial strains isolated from bathtub water were from family member. General features of each of the newly determined Bifidobacterium genomes

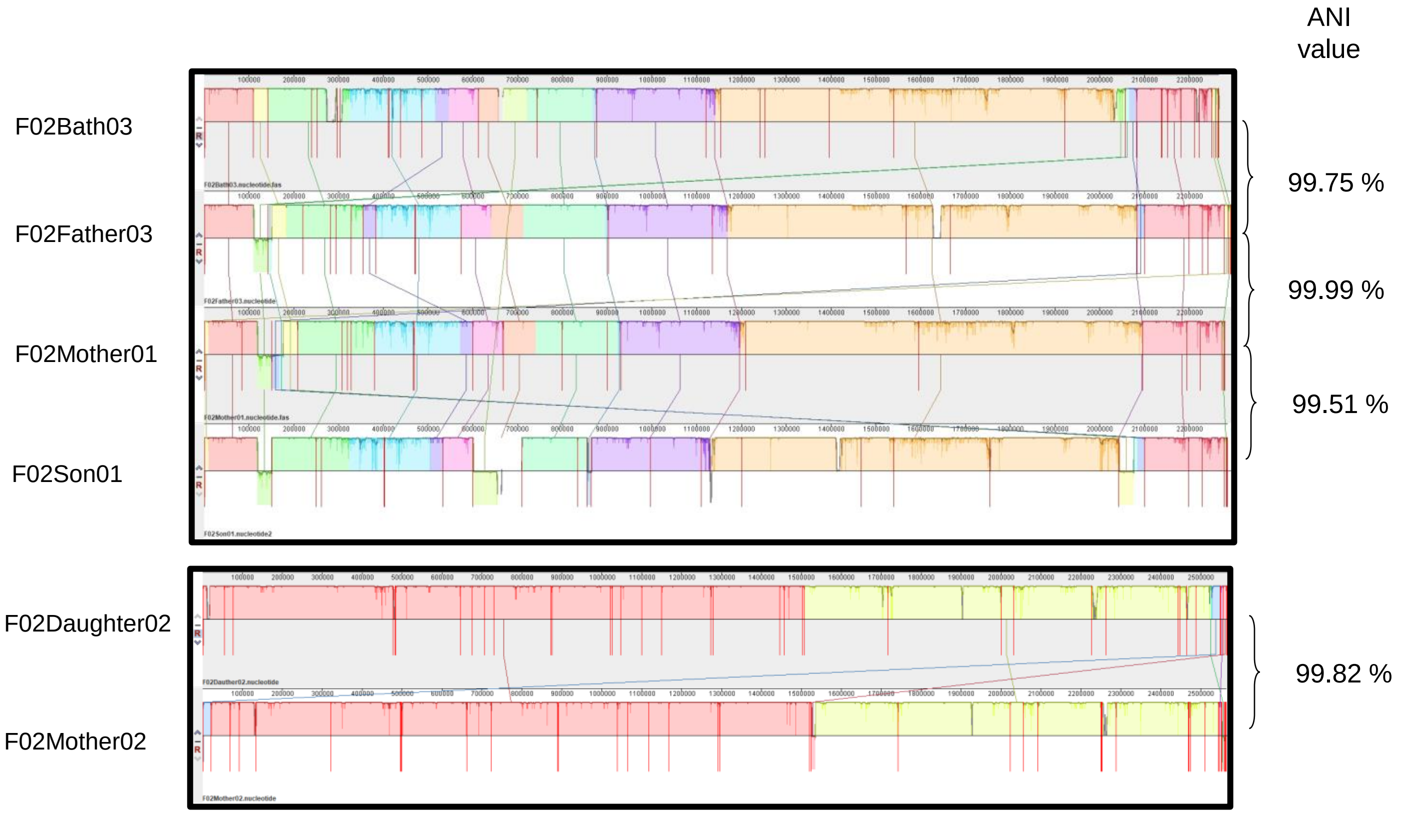

Supplementary Fig. 2b
Sequence similarity between strains putatively transmitted among family2 members and bathtub water
Related to Figure2. As shown in Mauve whole nucleotide alignment, certain strain-sets which were isolated from feces and bathtub water share near identical genome sequences, suggesting that bifidobacterial strains isolated from bathtub water were from family member. General features of each of the newly determined Bifidobacterium genomes

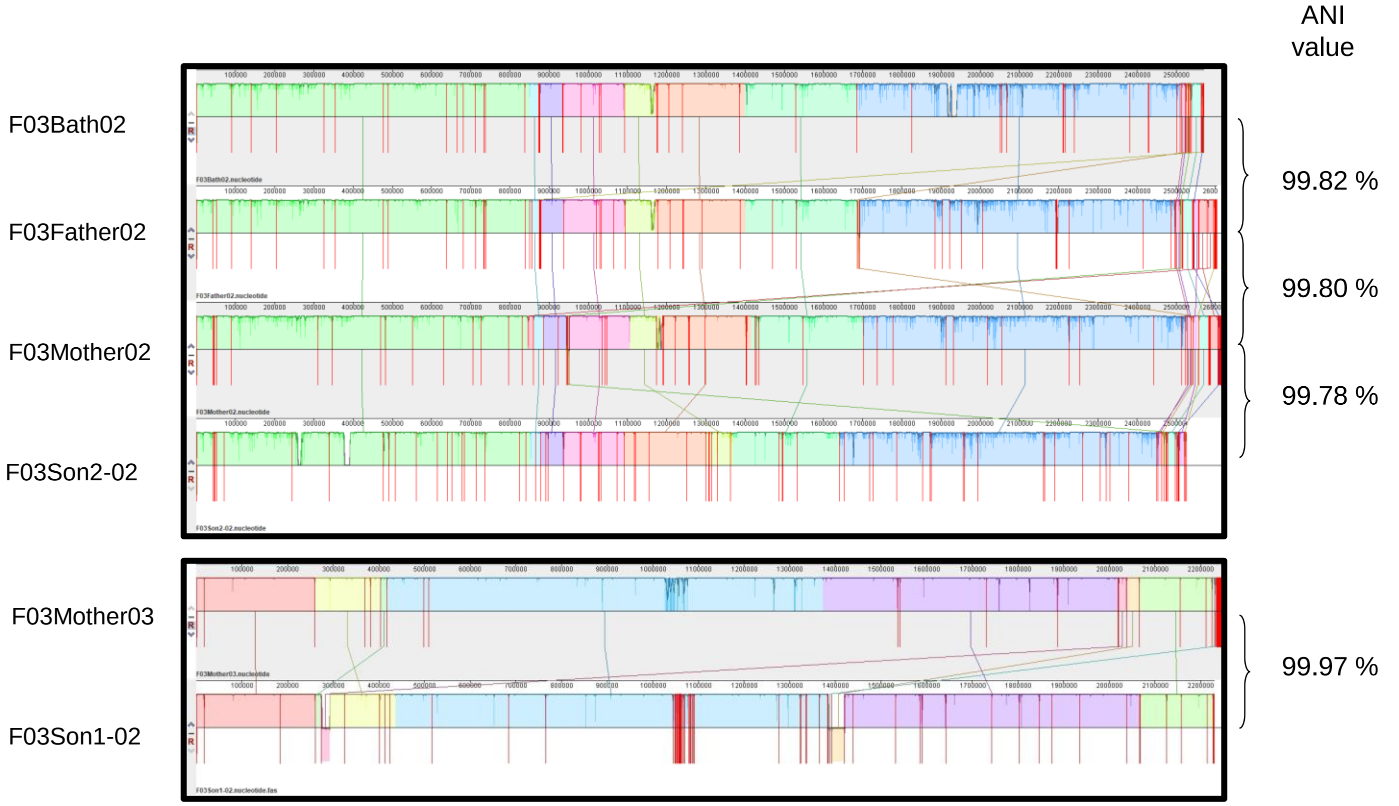

Supplementary Fig. 2c

Sequence similarity between strains putatively transmitted among family3 members and bathtub water

Related to Figure2. As shown in Mauve whole nucleotide alignment, certain strain-sets which were isolated from feces and bathtub water share near identical genome sequences, suggesting that bifidobacterial strains isolated from bathtub water were from family member. General features of each of the newly determined Bifidobacterium genomes

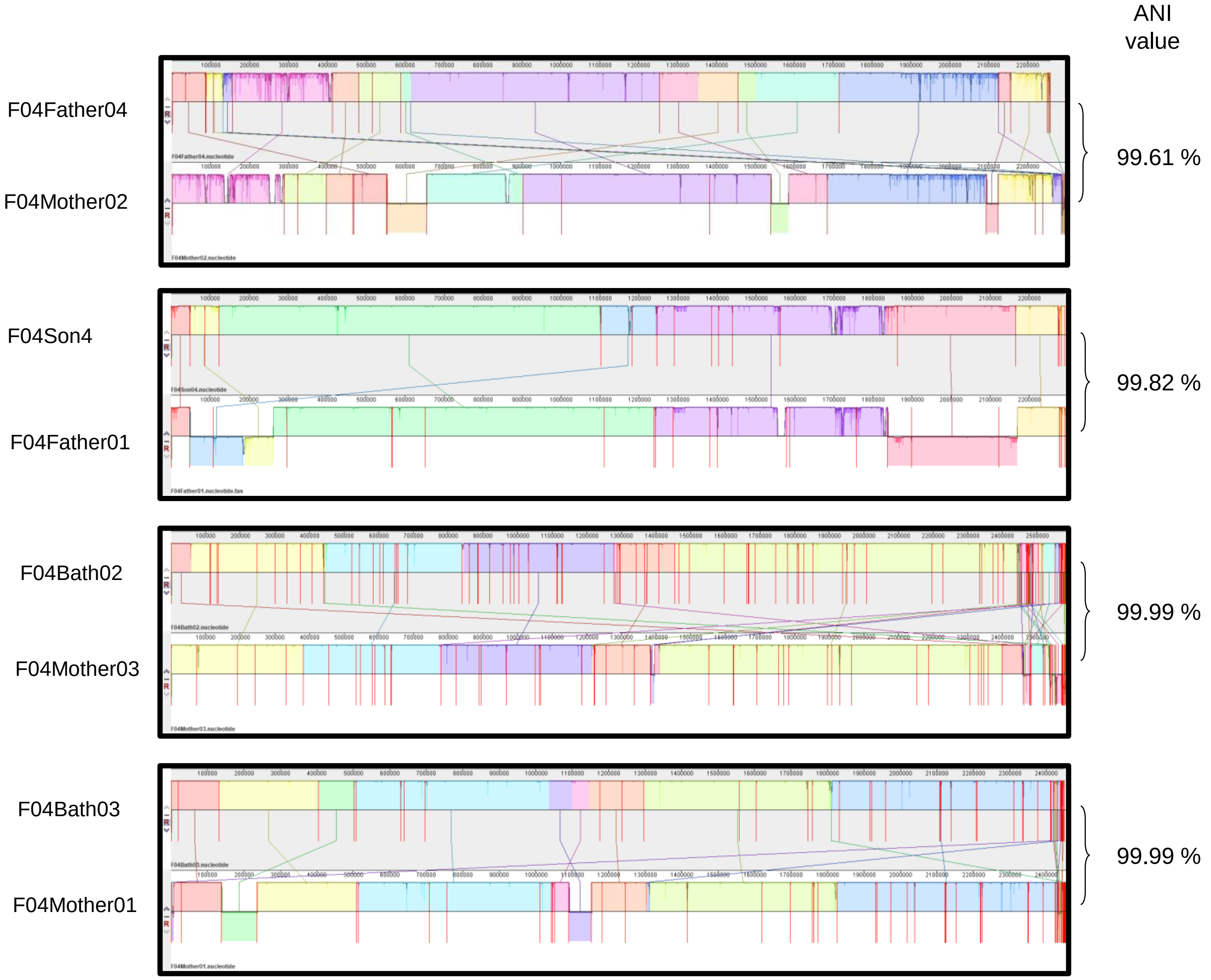

Supplementary Fig. 2d

Sequence similarity between strains putatively transmitted among family4 members and bathtub water

Related to Figure2. As shown in Mauve whole nucleotide alignment, certain strain-sets which were isolated from feces and bathtub water share near identical genome sequences, suggesting that bifidobacterial strains isolated from bathtub water were from family member. General features of each of the newly determined Bifidobacterium genomes

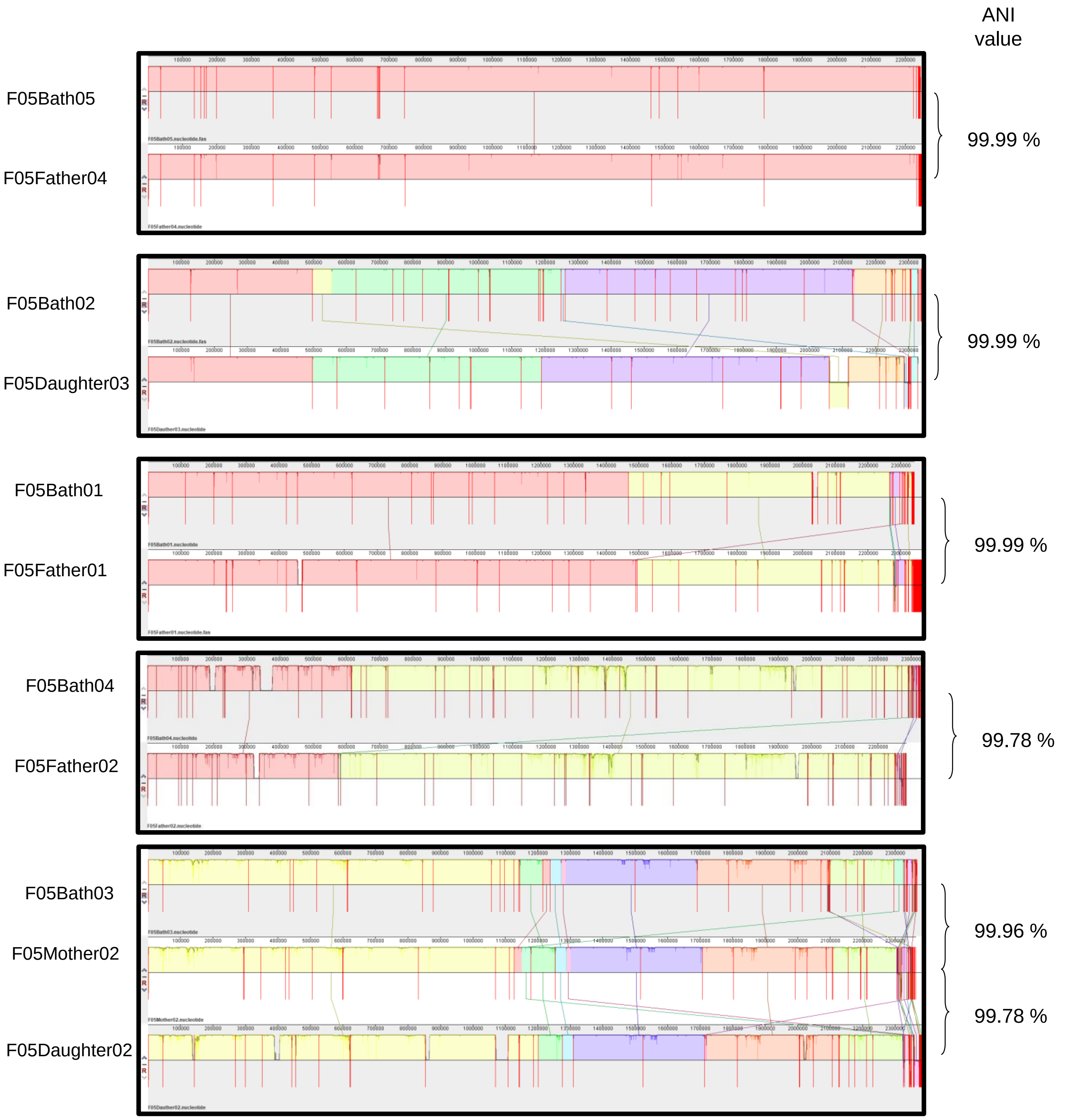

Supplementary Fig. 2e

Sequence similarity between strains putatively transmitted among family5 members and bathtub water

Related to Figure2. As shown in Mauve whole nucleotide alignment, certain strain-sets which were isolated from feces and bathtub water share near identical genome sequences, suggesting that bifidobacterial strains isolated from bathtub water were from family member. General features of each of the newly determined Bifidobacterium genomes
